# Substantial Dysregulation of miRNA Passenger Strands Underlies the Vascular Response to Injury

**DOI:** 10.3390/cells8020083

**Published:** 2019-01-23

**Authors:** Karine Pinel, Louise A. Diver, Katie White, Robert A. McDonald, Andrew H. Baker

**Affiliations:** Institute of Cardiovascular and Medical Sciences, BHF Glasgow Cardiovascular Research Centre, University of Glasgow, Glasgow G12 8TA, UK; drlouisediver@gmail.com (L.A.D.); kt_m_white@hotmail.com (K.W.); robertamcdonald8@gmail.com (R.A.M.)

**Keywords:** miRNA expression and regulation, passenger miRNA, biomarker, vascular injury, smooth muscle cells, porcine vein graft and stent models

## Abstract

Vascular smooth muscle cell (VSMC) dedifferentiation is a common feature of vascular disorders leading to pro-migratory and proliferative phenotypes, a process induced through growth factor and cytokine signaling cascades. Recently, many studies have demonstrated that small non-coding RNAs (miRNAs) can induce phenotypic effects on VSMCs in response to vessel injury. However, most studies have focused on the contribution of individual miRNAs. Our study aimed to conduct a detailed and unbiased analysis of both guide and passenger miRNA expression in vascular cells in vitro and disease models in vivo. We analyzed 100 miRNA stem loops by TaqMan Low Density Array (TLDA) from primary VSMCs in vitro. Intriguingly, we found that a larger proportion of the passenger strands was significantly dysregulated compared to the guide strands after exposure to pathological stimuli, such as platelet-derived growth factor (PDGF) and IL-1α. Similar findings were observed in response to injury in porcine vein grafts and stent models in vivo. In these studies, we reveal that the miRNA passenger strands are predominantly dysregulated in response to vascular injury.

## 1. Introduction

MicroRNAs (miRNAs) are a class of non-coding RNAs known to play a prominent role in gene regulation at a post-transcriptional level [1,2,3]. Pri-miRNAs are transcribed by RNA polymerase II and are processed successively by two RNase III enzymes, Drosha and Dicer, to a ~22 nt miRNA:miRNA * duplex [4], which, on processing, generates two single-stranded RNA molecules. miRNAs are loaded onto Argonaute to produce a RNA-induced silencing complex (RISC), which exerts translational repression via an imperfect binding to the mRNA target, generally localized in the 3′UTR. An equal amount of the two strands from the miRNA:miRNA * duplex is produced by the transcription but their accumulation becomes asymmetric [5]. Current miRNA nomenclature provides information on the direction of the mature miRNA strand using the -3p or -5p suffixes and, normally, the miRNA guide strand is thought to be the strand that preferentially accumulates in the RISC and the miRNA * or passenger strand (its partner) is degraded [6]. However, recent studies suggest that these passenger strands can accumulate in a number of disease pathologies and mediate strand-specific roles based on their distinct seed sequences and targets. Indeed, the mature sequences of the two strand forms can regulate distinct sets of mRNA transcripts or common targets through different hybridization sites on the mRNA targeted. 

miRNAs have been shown to induce phenotypic effects on smooth muscle cells (SMCs) in vascular disorders. Vascular SMC (VSMCs) within the vessel wall perform both contractile and proliferative functions in response to diverse cellular stimuli [7]. Under normal physiological conditions, VSMCs are relatively quiescent, harboring a contractile phenotype responding to changes in vascular flow to mediate relaxation and contraction. However, vascular injury or insult results in the initiation of phenotypic switching to a dedifferentiated phenotype. Indeed, the transition of VSMCs from a differentiated to a dedifferentiated phenotype is a common feature of vascular disorders where VSMCs harbor pro-proliferative and pro-migratory status. The VSMC phenotype can be modulated by many environmental cues and triggers, including a number of cytokines and growth factors released in response to injury. Indeed, platelet-derived growth factor (PDGF) promotes VSMC proliferation and migration resulting in neointimal formation after artery injury [8,9]. 

Since there is some recent evidence of the importance of the miRNA passenger strands [10,11,12], their individual functions have been studied in settings such as vascular cells with miR-126-5p and miR-10A*, [13,14] and cancer [15,16]. However, a more detailed or global evaluation of the importance of the miRNA passenger strands in vascular remodeling is still lacking. As miRNAs emerge as potential prognostic biomarkers and as attractive targets for disease intervention, we utilized array technology to evaluate the global expression of miRNA guide and passenger strands from hairpin precursors in pre-clinical porcine models of vascular injury and human VSMCs. 

## 2. Materials and Methods

### 2.1. Animals and Tissues

All the procedures for surgery, for the pig vein graft model and the stent model, were performed in reference [17] and [18], respectively, and were performed in accordance with the UK Home Office Guidance on the Operation of the Animals (Scientific Procedures) Act 1986 and with the institutional ethical approval (PPL60/4114 and PPL 60/4429). Male white Landrace pigs (SAC Commercial Ltd, Edinburgh, UK) were maintained on a 12 h light/dark cycle with free access to food and water at a designated Biological Procedures Unit. Briefly, the animals (weighing 19–26 kg) were pre-dosed orally with aspirin and clopidogrel 24 h prior to surgery. The animals were sedated by intramuscular (IM) injection of Tiletamine/Zolazepam (Zoletil; Virbac, France); general anesthesia was conducted using intravenous (IV) Propofol (Rapinovet; Schering-Plough, Welwyn Garden City, UK) and maintained with isoflurane (Abbott Laboratories Ltd, Berkshire, UK). Animals received 100 IU/kg of IV heparin (Leo Laboratories, London, UK) before surgery. After vascular access, coronary angiography was performed prior to the deployment of either bare metal stents (BMS) (Gazelle™, Biosensors, Morges, Switzerland) or drug eluting stents (DES) (biolimus A9 eluting stents; Biomatrix Flex™, Biosensors, Hägglingen, Switzerland). The unstented animals were used as controls. The pigs received 0.15 mg of buprenorphine IM (Vetergesic: Alstore Ltd, Sheriff Hutton, York, UK) and 350 mg of ampicillin IM (Amfipen LA, Intervet, UK) immediately following the procedure and were euthanized after 7 or 28 days by an intravenous overdose of euthatal, and their coronary vessels carefully removed. For the porcine vein graft surgery, following the induction with Ketaset (100 mg/mL ketamine hydrochloride), the animals were intubated and anesthetized with halothane and allowed to spontaneously ventilate. The method for the saphenous vein-carotid interpositional graft model has been described previously [19]. The saphenous vein was surgically exposed and harvested from the hind leg by a ‘no-touch’ technique. The vein was cannulated and gently irrigated with heparinized iso-osmotic sodium chloride (NaCl) solution (0.9 g/L). Each carotid was exposed via a longitudinal neck incision. The animal was heparinized by the IV administration of 100 IU/kg of heparin (CP Parmaceuticals Ltd, Wrexham, UK). A 4–5 cm segment of the carotid artery was isolated between the vascular clamps and 10 mm was then removed. The residual ends were beveled at 45° and a small longitudinal incision was made to lengthen the anastomotic area. The ends of the vein were similarly beveled and anastomosed as an interposition graft under optical magnification using continuous 7/0 Surgipro (auto Suture, Dagford, UK) sutures. The saphenous veins from the ungrafted animals were used as controls. The animals were recovered, returned to their pen and maintained on a normal chow diet for the duration of the experiment.

Surplus human saphenous vein tissue was obtained with informed consent from patients undergoing CABG (Coronary Artery Bypass Grafting). All procedures received local ethical approval (Research Ethical Committee number: 06/S0703/110) and the experimental procedures conformed to the principles outlined in the Declaration of Helsinki.

### 2.2. Vessel Storage and RNA Isolation from the Pig Vein Graft Model and Stent Model

All the procedures for RNA extraction from the pig vein graft model and stent model are described in detail in [17,18], respectively. Briefly, the harvested vessels were placed in RNAlater^®^-ice (Invitrogen, Paisley, UK) and stored at −80 °C until the day of isolation. RNAs from the veins or arteries were isolated following disruption of the vessels under liquid nitrogen using a pestle and mortar, and these vessel fragments were placed in Qiazol (Qiagen, Hilden, Germany) and homogenized using a tissue homogenizer (Polytron, Switzerland). The RNAs were processed through miRNEasy Mini Kit (Qiagen) following the manufacturer’s instructions, treated with DNAse 1 (amplification grade; Sigma, St. Louis, MO, USA) in order to eliminate genomic DNA contamination and quantified using a NanoDrop ND-1000 Spectrophotometer (Nano-Drop Technologies, Wilmington, DE, USA). RNA integrity was assessed using the RNA 6000 Nano LabChip kit (Agilent Technologies, Santa Clara, CA, USA). Only the RNAs with an RNA integrity number value >7 were used for the TLDA experiments.

### 2.3. Cell Culture

VSMCs were isolated from human saphenous vein segments within 24 h of surgery using the explant technique as previously described by Southgate et al. [20]. Briefly, following removal of the adventitial layer using sterile forceps and scissors, the vein was longitudinally opened. The lumenal surface of the vein was scraped gently with a rubber policeman to remove the endothelium. Using fine forceps, thin segments of the medial layer were stripped from the vein. The stripped medial segments were then cut into approximately 1 mm^2^ pieces using a tissue chopper and then transferred into a sterile tube containing wash media (DMEM medium supplemented with 2 mmol/L of l-glutamine (Invitrogen), 50 µg/mL of penicillin (Life Technologies, Paisley, UK) and 50 µg/L of streptomycin (Life Technologies)). Following two washes in the wash media, the segments were resuspended in a small volume of SMC complete medium (SMC growth medium 2 (PromoCell, Heidelberg, Germany) supplemented with 15% foetal calf serum (FCS) (PAA laboratories, Yeovil, UK), 2 mmol/L of l-glutamine (Invitrogen), 50 µg/mL of penicillin (Life Technologies) and 50 µg/L of streptomycin (Life Technologies)) and were then spread onto the base of 25 cm^2^ tissue culture flasks. Following overnight culture at 37 °C in a humidified atmosphere containing 5% CO_2_ (*v*/*v*), 5 mL of SMC complete medium was carefully added to each flask. The media were changed every 3–5 days and after 2–3 weeks, the outgrowth of VSMCs was passaged into 75 cm^2^ flasks and grown to confluence. All experiments were performed using VSMCs between passages 3 and 6. The VSMCs were plated in SMC complete medium, quiesced in medium (DMEM medium supplemented with 2 mmol/L of l-glutamine (Invitrogen), 50 µg/mL of penicillin (Life Technologies) and 50 µg/L of streptomycin (Life Technologies)) containing 0.2% FCS for 48 h. The plated cells were either placed in fresh 0.2% FCS medium (control) or stimulated with 0.2% medium supplemented with PDGF (PDGF-BB, 20 ng/mL) or with IL-1α (10 ng/mL) or a combination of PDGF and IL-1α for 48 h before RNA isolation.

### 2.4. RNA Extraction and Purification

For the extraction of RNAs from VSMCs, the lysis of the plated cells was performed with 700 µL of QIAzol lysis reagent (Qiagen). The purification of total RNA was achieved using miRNEasy Mini Kit (Qiagen) according to the manufacturer’s instructions. The RNA integrity and concentration were determined using the NanoDrop ND-1000 spectrophotometer.

### 2.5. TaqMan Low Density Array (TLDA) Experiment

miRNA profiling was performed with the Human MicroRNA Array v2.0 Card A and B which is enabled to quantify 667 human miRNAs catalogued in the miRBase v10. For three independent patients (Pt 1, Pt 2 and Pt 3), 90 ng of total RNA was reverse transcribed using the Megaplex RT set pool A and B and then preamplified following the manufacturer’s instructions (Applied Biosystems, Carlsbad, CA, USA). The cDNA that was produced was loaded on the Human MicroRNA Array cards and run on the 7900HT Fast Real Time PCR system (Applied Biosystems) using SDS2.3 software. Data analysis was performed using DataAssist v2.0 Software (Applied Biosystems). The miRNA expression data were normalized using the RNU48 housekeeper expression because of their low Ct variance. 

The TLDA experiments for the pig vein graft model and the pig stent model are described in detail in ref [17] and [18], respectively.

### 2.6. Global Analysis

The literature and databases such as the miRBase Sequence Database (Release 21) permit the determination of the guide and the passenger strands for a thousand stem loops from the data obtained by the TLDA experiments. The level of expression (relative expression, %) was classified into three groups: high (Ct < 23), medium (Ct between 23 and 28) and low (Ct > 28) expressions. The dysregulated miRNAs are represented by a relative quotient (RQ) higher than two or less than 0.5 in expression level.

### 2.7. Gene Expression Quantitative RealTime-PCR (qRT-PCR)

The cDNA for gene expression analysis was synthesized from 400 ng of RNA using TaqMan Reverse Transcription Reagents (Applied Biosystems). The reverse transcription (RT) reactions were carried out in a volume of 20 μL following the manufacturer’s instructions and run in a thermocycler under the conditions: 25 °C for 10 min, 48 °C for 30 min and 95 °C for 5 min, then held at 4 °C. 

The TaqMan real-time PCR reactions were performed in triplicate comprising 5 μL of TaqMan Universal Master Mix II, no UNG (Applied Biosystems), 0.5 μL of 20× gene-specific primer and a probe mix (TaqMan gene expression assays, Cat number # 4331182 (CDKN1B, Assay ID: Hs01597588_m1; UBC, Assay ID: Hs00824723_m1)), 1.2 μL of the reverse transcription product and 3.3 µL of nuclease free water. RT-PCR was carried out by using a 7900HT Fast Real Time PCR system (Applied Biosystems) under the conditions: 50 °C for 2 min, 95 °C for 10 min, and then 40 cycles of 95 °C for 15 s, 60 °C for 60 s, then held at 4 °C. The gene expression level was normalized to UBC (Ubiquitin C) and the fold-changes were calculated using the ΔΔCT method.

### 2.8. miRNA Expression Quantitative RealTime-PCR (qRT-PCR)

All the miRNA specific probes (TaqMan microRNA assays) are commercial and available from Life Technologies: Cat number # 4427975 (hsa-miR-204, Assay ID: 000508; hsa-miR-218, Assay ID: 000521; hsa-miR-1275, Assay ID: 002840; hsa-miR-625*, Assay ID: 002432; hsa-miR-222*, Assay ID: 002097). 

The RT reactions were performed using the TaqMan miRNA Reverse Transcription Kit (Applied Biosystems) and miRNA-specific RT stem-loop primers (Applied Biosystems). The RT reactions were carried out in a volume of 7.5 µL following the manufacturer’s instructions and run in a thermocycler under the conditions: 16 °C for 30 min, 42 °C for 45 min and 85 °C for 5 min, then held at 4 °C.

The TaqMan real-time PCR reactions were performed in triplicate comprising 5 µL TaqMan Universal Master Mix II, no UNG (Applied Biosystems), 0.5 µL of 20 × miRNA-specific primer and a probe mix of the TaqMan miRNA Assay Kit (Applied Biosystems), 0.7 µL of the RT product and 3.8 µL of nuclease free water. RT-PCR was carried out by using a 7900HT Fast Real Time PCR system (Applied Biosystems) under the conditions: 95 °C for 10 min, then 40 cycles of 95 °C for 15 s, 60 °C for 60 s, then held at 4 °C. The expression level of miRNA was normalized to RNU48 and the fold-changes were calculated using the ΔΔCT method. Only the cells from patients harboring a low variation concerning the baseline expression of miRNAs have been used for the miRNA expression analysis (Figure S1).

### 2.9. Statistical Analysis

The statistical analysis was performed according to figure legends. The statement N = 3 means that three experiments were performed, three independent times, all in triplicate. Data are expressed as mean ± SEM. Comparisons between the two groups were analyzed using the 2-tailed Student’s *t*-test. The one-way ANOVA with Tukey’s post hoc multiple comparison test, via Graph Pad Prism version 5.0 (GraphPad Software, San Diego, CA, USA), was used for comparisons among three or more groups. A statistical difference was considered as *p* < 0.05. Heat maps were generated using the R program (version 3.2.4) (R Foundation for Statistical Computing, Vienna, Austria).

## 3. Results

### 3.1. Global Analysis of miRNA in VSMCs in Response to Cytokine and Growth Factor Stimulation and in Vascular Injury In Vivo

**Figure 1:**
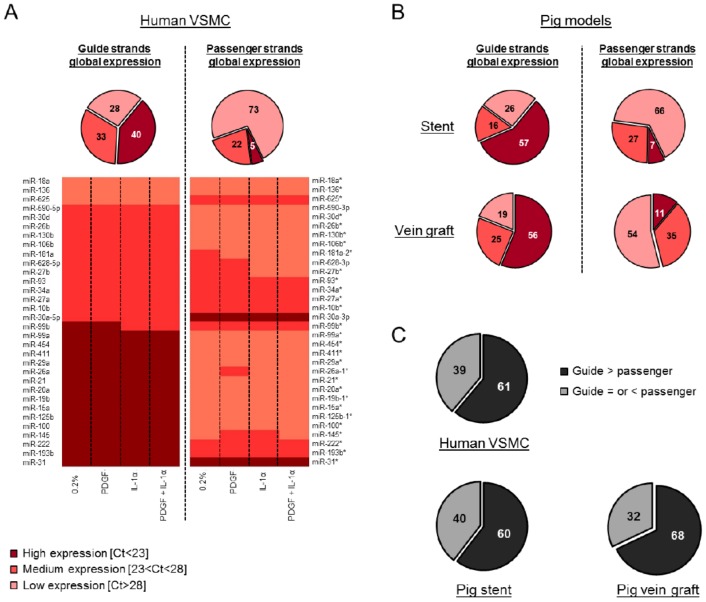
Low passenger strands expression compared to the guide strands in VSMCs and in vivo. One hundred hairpins were analyzed by TaqMan Low Density Array (TLDA) in human VSMCs (average of *n* = 3), the porcine models of vein graft failure and in stent restenosis (average of *n* = 6 for both the porcine models, except for the 7 days DES (drug eluting stent) condition, where *n* = 5). The level of expression (relative expression, %) was classified into three groups: high (Ct < 23), medium (Ct between 23 and 28) and low (Ct > 28) expressions. These groups are represented as a % in each pie chart. (**A**) Each pie chart represents the global expression of the guide and passenger strands in human VSMCs. The heat map illustrates the relative abundance of the number of miRNA hairpins that were consistently expressed across three independent patients and represents the level of expression of the guide versus passenger strands. (**B**) Each pie chart represents the global expression of the guide and passenger strands in the porcine model of in stent restenosis and vein graft failure. (**C**) Each pie chart represents the expression of the guide versus passenger strands within each independent hairpin.

Human VSMCs were treated with PDGF, interleukin 1α (IL-1α) or a combination of both to mimic a number of the pathologic signals induced by vascular injury. As expected, PDGF induced a significant increase in VSMC migration (Figure S2A), and the pro-proliferative phenotype was confirmed by a reduction in the expression of the cyclin-dependent kinase inhibitor 1B (CDKN1B) (Figure S2B). Following confirmation of the phenotypic alternation in VSMCs, we performed a detailed and unbiased analysis of both the guide and passenger miRNAs expression in VSMCs (Table S1). Indeed, using TaqMan Low Density Arrays (TLDA) we were able to quantify a large number of miRNAs simultaneously in each individual sample. The relative expression in each condition was compared to the unstimulated quiested VSMCs and expressed in fold change. Globally, our data highlighted a low variation between the samples. Bioinformatics tools including the miRBase (Realease 21) (http://www.mirbase.org/) have been used to discriminate “guide” versus “passenger” strands. We validated five miRNAs from our array by subsequent assays (Figure S3) and found miRNA markers in our global analysis such as miR-146a, an inflammation-associated miRNA [21], which showed overexpression following exposure to IL-1α. From our array, the level of expression was classified into three groups following pre-amplification according to cycle threshold (Ct) values (low [Ct > 28], medium [Ct between 23 and 28] and high [Ct < 23] expression) for each miRNA. The global analysis revealed that the miRNA guide strands are more abundantly expressed than the passenger strands (Figure 1A) in all the conditions tested (Figure S4A). A similar pattern was identified following the computational analysis of the array data from the porcine models of vein graft failure [17] and in stent restenosis [18] (Figure S4B and Figure S4C, respectively, Figure 1B and Figure S5). A total of one hundred hairpins were analyzed from the data generated by TLDA. Consistently across the three models, we found that the hairpin of miR-10b, miR-193b, miR-199a, miR-214, miR-30a, miR-335 and miR-99b contained medium or highly expressed passenger strands. Furthermore, a direct comparison of the relative expression of miRNA strands obtained from their corresponding hairpins confirmed that the guide strand was more abundantly expressed than its corresponding passenger strand in more than 60% of cases, a pattern that was consistent across all three data sets (Figure 1C). In human VSMCs, the guide strand appeared to be the most predominantly expressed variant of the hairpin, although there are exceptions where the passenger strands were more abundantly expressed than their corresponding guide strand such as miR-625 and miR-30a (heat map, Figure 1A). The same type of exception has been found across the three models for miR-199a, miR-30a, and miR-335. 

### 3.2. Passenger Strands Are Dysregulated More Frequently than Guide Strands in Vascular Cells and Vascular Injury In Vivo

**Figure 2:**
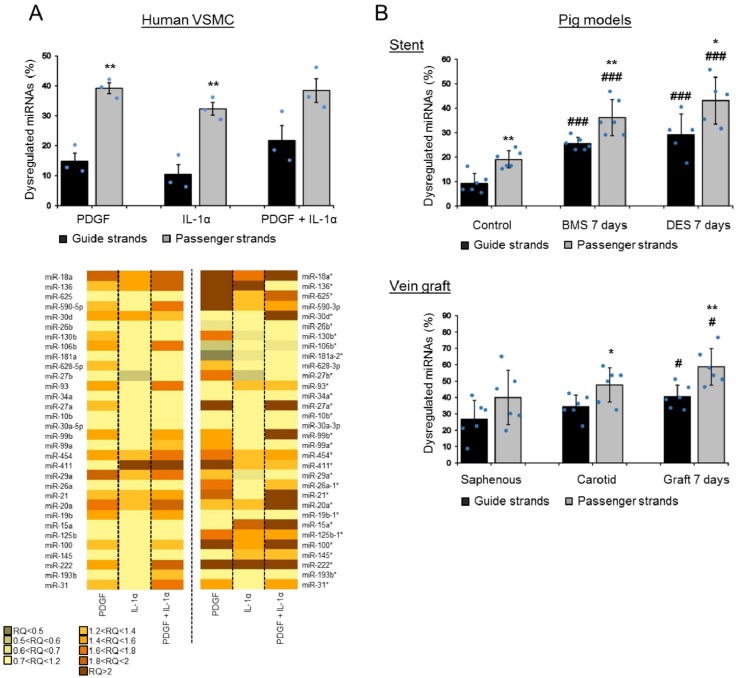
High passenger strand dysregulation compared to the guide strand dysregulation in VSMCs and in vivo. The graphs show the percentage of dysregulated miRNAs for each form in the three independent models. (**A**) The relative dysregulation of each strand of the miRNA hairpin was quantified after stimulation by platelet-derived growth factor (PDGF) and IL-1α in human VSMCs (the statistics have been made between the passenger and guide strands within each condition; unpaired *t*-test; ** *p* < 0.01 for passenger vs guide strands). The heat map illustrates the relative dysregulation (in relative quotient (RQ)) of a number of miRNA hairpins after stimulation by PDGF and IL-1α across three independent patient samples (average of *n* = 3). (**B**) The dysregulation of the guide and passenger strands was quantified at 7 days following stenting (BMS (bare-metal stent) or DES (drug eluting stent)) or at 7 days following vein grafting in vivo (*n* = 6 for both the porcine models, except for the 7 days in the DES condition where *n* = 5) (the statistics have been made between the passenger and guide strands within each condition; unpaired *t*-test; * *p* < 0.05 and ** *p* < 0.01; and vs. the corresponding control (unstented control animals or saphenous), # *p* < 0.05 and ### *p* < 0.001).

Despite their generally lower levels of expression compared to the guide strands, we then quantified the dysregulation of the passenger strands in vascular cells in vitro and in injury models in vivo. Interestingly, in all three settings, we found a substantial and predominant dysregulation of the passenger strands in response to pathological stimuli (Figure 2). The relative number of dysregulated miRNAs included 39% and 32% of the passenger strands and only 15% and 10% of the guide strands in human VSMCs treated with PDGF and IL-1α, respectively (Figure 2A). Furthermore, all together, 20% of the dysregulation concerned the two strands in a hairpin. In vivo, 36% and 43% of the passenger strands were dysregulated compared to 25% and 29% for the guide strands at 7 days following stenting by BMS (bare-metal stent) and DES (drug eluting stent), respectively (Figure 2B). This dysregulation was still at 32% and 39% for the passenger strands compared to 19% and 23% for the guide strands at 28 days following stenting by BMS and DES, respectively (Figure S6A). In our vein graft failure model, we found 59% of the passenger strands dysregulated compared to 40% the guide strands at 7 days following engraftment (Figure 2B). This dysregulation reached 68% for the passenger strands and 33% for the guide strands at 28 days (Figure S6B). Additionally, all together, we found 47% and 31% of the dysregulation concerned the two strands in a hairpin in the pig vein graft model and in the pig stent model, respectively. 

## 4. Discussion

VSMCs are typically quiescent and contractile under normal physiological conditions. However, following vascular injury, the release of cytokines and growth factors initiates a transcriptional cascade resulting in the initiation of a process called “phenotypic switching” and this leads the VSMCs to harbor a synthetic, pro-proliferative and pro-migratory state. Since miRNAs regulate mRNA expression and thereby many fundamental biological processes, they are intensely studied as candidates for diagnostic and prognostic biomarkers in a number of diseases. Indeed, miRNAs have been shown to play important roles in many cellular processes such as cell differentiation, proliferation and migration. We decided to conduct a detailed analysis in order to understand the microRNAome through TLDA in the pre-clinical porcine models of vascular injury and human VSMCs. It is important to note that we utilized human arrays in the porcine samples to try and identify a miRNA which would extrapolate to the pathology in the clinical setting, so it is possible that a proportion of miRNAs may be under-represented due to the sequence variation or chromosomal locations between pig and human. Our global analysis shows the dysregulation of a number of miRNAs already described in the literature as phenotypic regulators of VSMCs. For example, we found that miR-222, previously reported to be involved in vascular pathologies associated with excessive rates of SMC proliferation and migration [22], was substantially up-regulated in response to pathological stimuli. Indeed, in our arrays, miR-222 was overexpressed upon PDGF stimulation in VSMCs (Figure 2 and Figure S3) as well as following DES implantation and vein grafting in the porcine models (Figure S6). Furthermore, we found that miR-18a was upregulated following vein grafting and stenting in pig models (Figure S6). This finding is in accordance with the literature, in which miR-18a has been found to be upregulated after rat carotid balloon injury and to promote VSMC differentiation [23]. Furthermore, miR-145 was downregulated following vein grafting and during the early stage after stenting in our porcine models (Figure S6). MiR-145 is extensively studied in vascular biology for its ability to control vascular neointimal lesion formation. Indeed, the restoration of miR-145 in balloon-injured arteries inhibits neointimal growth in rat [24]. Moreover, our global analysis gave a global landscape of the miRNA guide strand as well as passenger strand expression. Indeed, recent studies suggest that these passenger strands can mediate strand-specific roles based on their distinct seed sequences and targets, highlighting their importance [13,14]. We decided to study the behavior of miRNA depending on the strand form after exposure to pathological stimuli via our detailed analysis of the miRNA transcriptome through TLDA. Our results confirmed a higher global expression of the guide strands compared to passenger strands. Few exceptions with a higher expression of the passenger strands of the miRNA:miRNA * duplex have been noted, and this suggests the possibility that they can mediate functional effects on mRNA targets. However, our study is the first to show that a greater proportion of passenger strand miRNAs was significantly altered after exposure to the pathological mediators of vascular remodeling, PDGF and/or IL-1α in vitro and post-stenting or grafting in vivo. Some studies reported a preferred arm switched between samples and tissues [25,26]. In our study, after vascular injury, the selection of the mature miRNA strand via an “arm switching” mechanism could be responsible for the greater proportion of the dysregulated passenger strands that was obtained. It is widely accepted that vascular injury is responsible for the “phenotypic switching” induction in VSMCs but our study suggests that vascular injury could also be implicated in the aforementioned miRNA “arm switching” phenomenon. 

In conclusion, we demonstrated via a global analysis that the miRNA passenger strands are frequently dysregulated in acute vascular injury. Our detailed and unbiased analysis emphasized an interesting landscape of miRNAs in vascular biology where the passenger strands contribute more broadly than previously thought and may be attractive targets for disease intervention.

## Supplementary Materials

The following are available online, Figure S1: Low variation of the baseline miRNAs expression, Figure S2: Validation of VSMC stimulation, Figure S3: Validation of TLDA arrays expression profiles by qRT-PCR, Figure S4: Higher expression of the guide strands compared to the passenger strands in VSMCs and in vivo, Figure S5: Low passenger strands expression compared to the guide strands in stent and vein graft porcine models, Figure S6: High passenger strands dysregulation compared to the guide strands 28 days following stenting or grafting in porcine models, Table S1: miRNAs expression analyzed by TLDA in human VSMCs.
