# Supplementary material for: Substantial Dysregulation of miRNA Passenger Strands Underlies the Vascular Response to Injury
Source: Cells. 2019 Jan 23;8(2):83. doi: 10.3390/cells8020083 (PMC6406808; doi:10.3390/cells8020083)
Supplement: Supplementary file 1 [file cells-08-00083-s001.zip › Pinel-Supplementary-files/Pinel-Supplementary information.docx]

**Supplemental Figure Legends**

**Figure S1. Low variation of the baseline miRNAs expression.** Baseline expression (0.2% FCS) for miR-204, miR-218, miR-1275 and miR-625* in all primary VSMC from patients used for TLDA validation (n=3).

**Figure S2. Validation of VSMC stimulation.** (**A**) PDGF induce VSMC migration. Scratch injury was performed on starved cells either placed in 0.2% FCS medium or 0.2% FCS medium supplemented with PDGF. VSMC migration was monitored 14 h following scratch injury (n=3) (unpaired t-test; **P<0.01: vs 0.2%). The wounds were photographed using a 10x objective (scale bar represent 100 µm). (**B**) CDKN1B down-regulation following stimulation of quiescent VSMC for a period of 48 h with PDGF, IL-1α or a combination of both (n=3) (one-way ANOVA, ****P<0.0001: vs 0.2% FCS; ##P<0.01 and ###P<0.001: vs IL-1α).

**Figure S3. Validation of TLDA arrays expression profiles by qRT-PCR.** TLDA measurements and validations by qRT-PCR of 5 independent miRNAs: miR-204, miR-218, miR-1275, miR-625* (miR-625-3p) and miR-222* (miR-222-5p), following VSMC stimulation during 48 h with PDGF, IL-1α or a combination for 3 independent patients (Pt1, Pt2 and Pt3) (one-way ANOVA; *P<0.05, **P<0.01, ***P<0.001 and ****P<0.0001: vs 0.2% FCS; #P<0.05, ##P<0.01, ###P<0.001: vs IL-1α).

**Figure S4. Higher expression of the guide stands compared to the passenger strands in VSMCs and in vivo*.*** One hundred hairpins were analysed by TLDA. (**A**) Analysis in human VSMC (average of n=3) (statistics have been made between passenger and guide strands for each condition; unpaired t-test; *P<0.05). (**B**) Analysis in porcine models of vein graft failure (average of n=6) (statistics have been made between passenger and guide strands for each condition; unpaired t-test; ***P<0.001). (**C**) Analysis in in stent restenosis model (average of n=6, excepted for the 7 days DES condition where n=5) (statistics have been made between passenger and guide strands for each condition; unpaired t-test; ***P<0.001).

**Figure S5. Low passenger strands expression compared to the guide strands in stent and vein graft porcine models.** The heat map illustrates the relative abundance of a number of miRNAs hairpins that were consistently expressed across the animals and represent the level of expression of the guide versus passenger strands. The level of expression was classified into 3 groups; high (Ct<23), medium (Ct between 23 and 28) and low (Ct>28) expressions.

**Figure S6. High passenger strands dysregulation compared to the guide strands 28 days following stenting or grafting in porcine models.** (**A**) The dysregulation of guide and passenger strand was quantified 28 days following stenting (BMS or DES) *in vivo* (n=6) (unpaired t-test; *P<0.05 and ***P<0.001 for passenger vs guide strands for each condition; #P<0.05 and ##P<0.01: vs corresponding control). The heat map illustrates the relative dysregulation of a number of miRNAs in all conditions, 7 days and 28 days after stenting (average of n=6, excepted for the 7 days DES condition where n=5). (**B**) The dysregulation of guide and passenger strand was quantified 28 days following vein grafting *in vivo* (n=6) (unpaired t-test; *P<0.05, **P<0.01 and ***P<0.001 for passenger vs guide strands for each condition; #P<0.05 and ##P<0.01: vs Saphenous). The heat map illustrates the relative dysregulation of a number of miRNAs in all conditions, 7 days and 28 days after vein grafting (average of n=6).

**Supplemental Table Legends**

**Table S1. MiRNAs expression analyzed by TLDA in human VSMCs.** MiRNAs fold changes following 48 h stimulation of VSMC with PDGF, IL-1α or a combination of both (n=3). TLDA analysis was performed using Human MicroRNA Array v2.0 Card A and B.

**Supplemental Methods**

**Migration assay**

VSMC migration was analyzed by scratch wound assay. Cells quiesced for 48 h were either placed in 0.2% FCS medium or 0.2% FCS medium supplemented with PDGF and then wounded with a sterile 20 μL pipette tip in a predetermined grid pattern and the medium was renewed. The wounds were photographed at baseline and 14 h later using an ENOS XL Core (Life Technologies) camera system using a 10x objective.
