# Supplementary figures and images for: Substantial Dysregulation of miRNA Passenger Strands Underlies the Vascular Response to Injury
Source: Cells. 2019 Jan 23;8(2):83. doi: 10.3390/cells8020083 (PMC6406808; doi:10.3390/cells8020083)

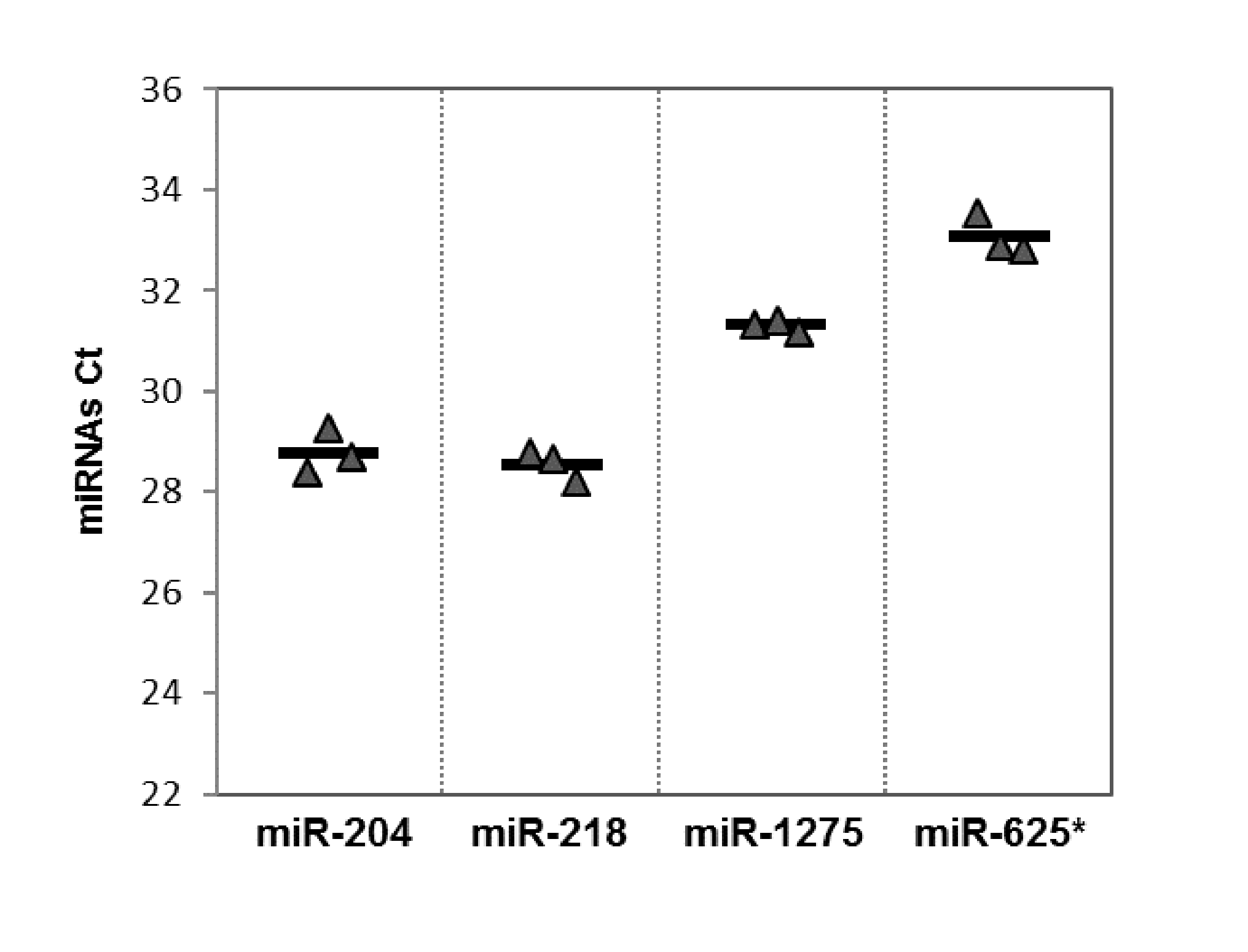

Supplement: Supplementary file 1 [file cells-08-00083-s001.zip › Pinel-Supplementary-files/Pinel-FigureS1.png]

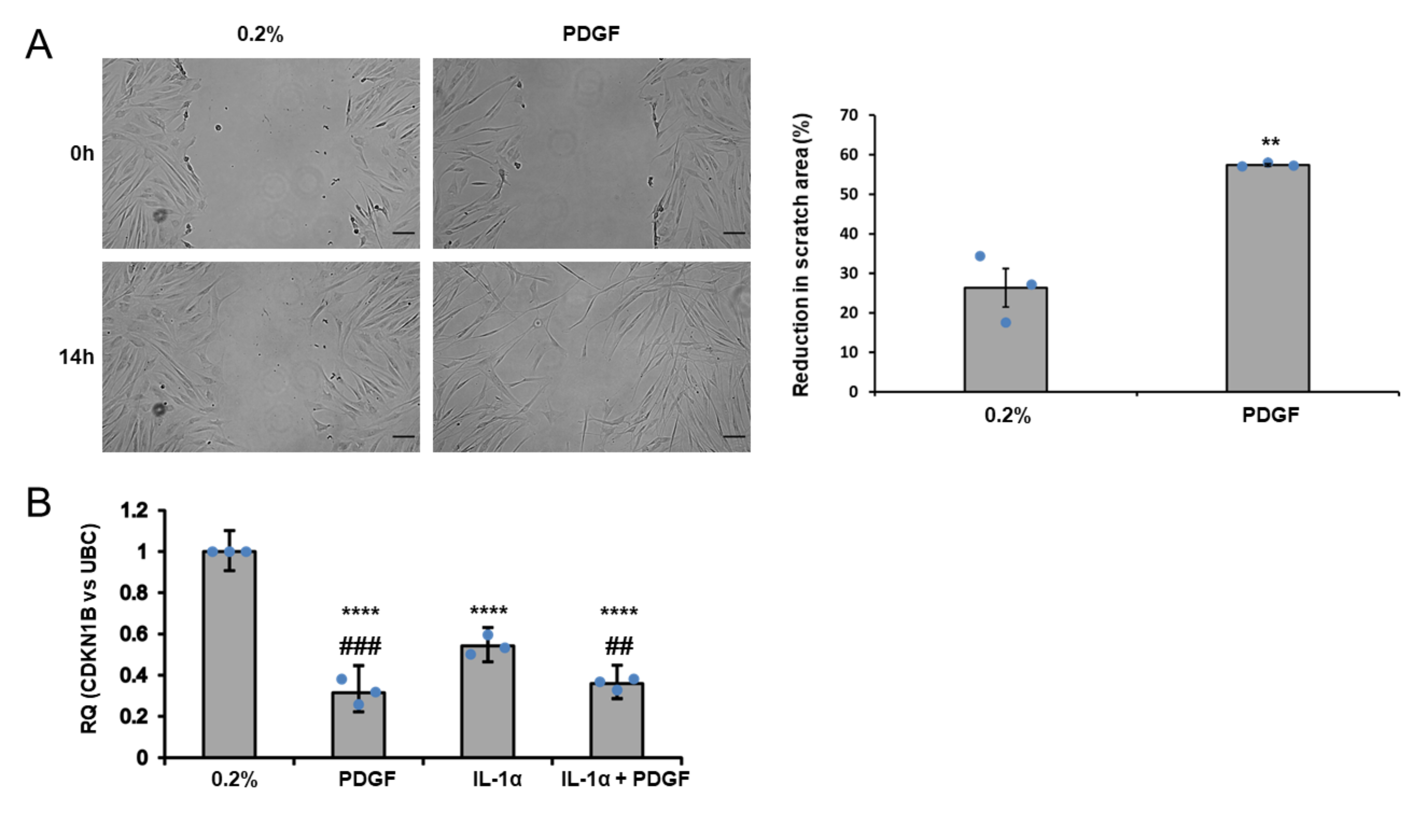

Supplement: Supplementary file 1 [file cells-08-00083-s001.zip › Pinel-Supplementary-files/Pinel-FigureS2.png]

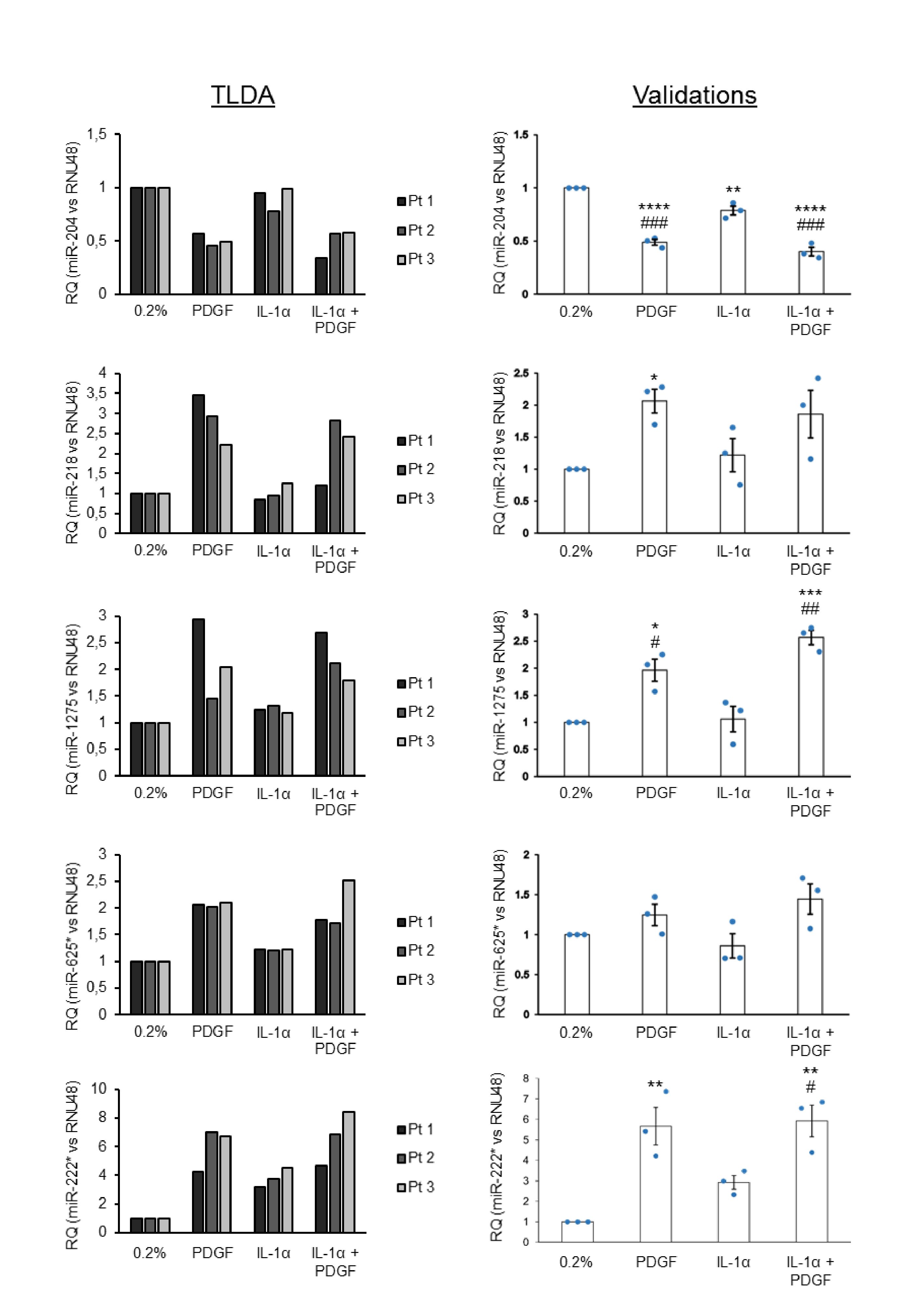

Supplement: Supplementary file 1 [file cells-08-00083-s001.zip › Pinel-Supplementary-files/Pinel-FigureS3.png]

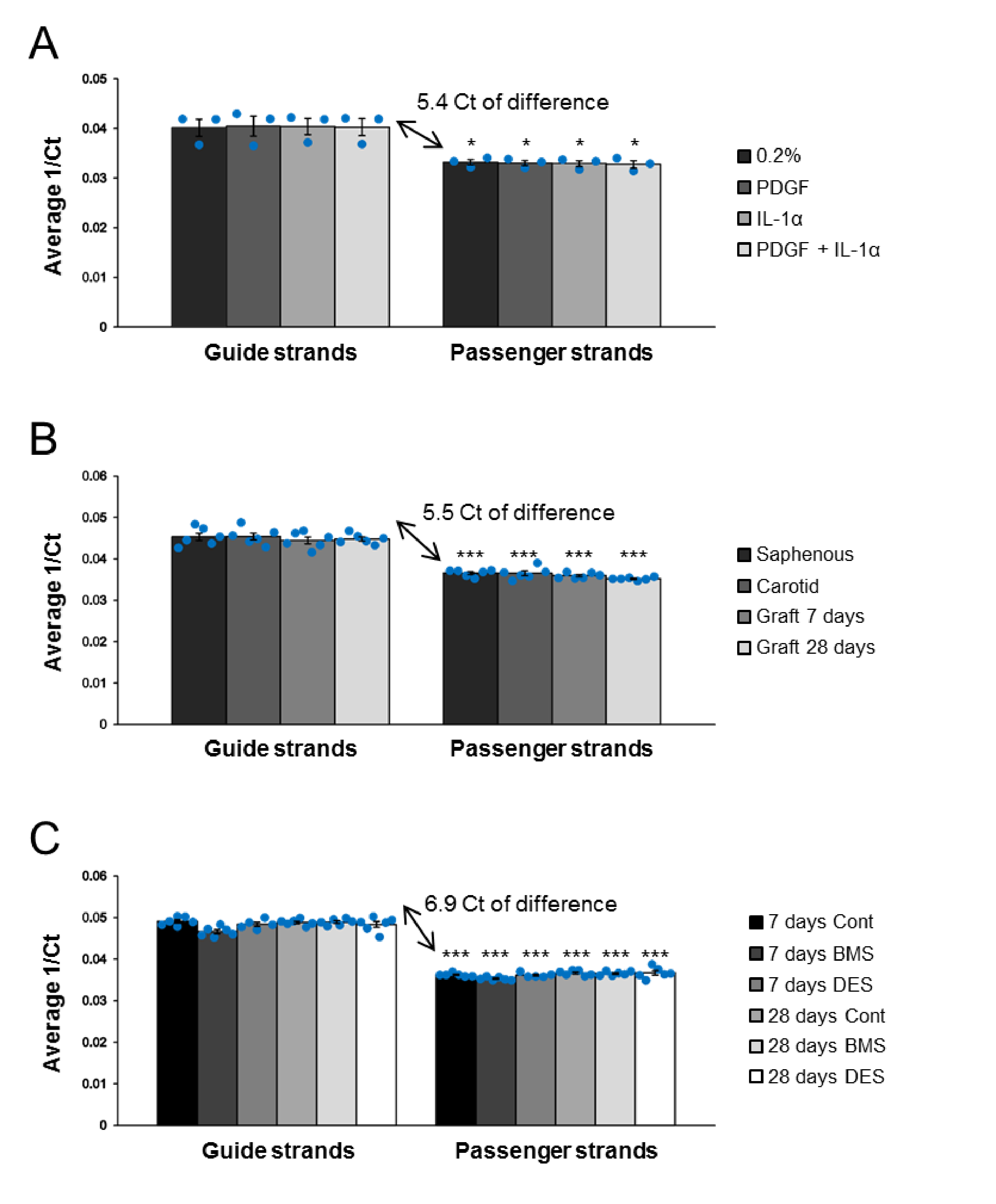

Supplement: Supplementary file 1 [file cells-08-00083-s001.zip › Pinel-Supplementary-files/Pinel-FigureS4.png]

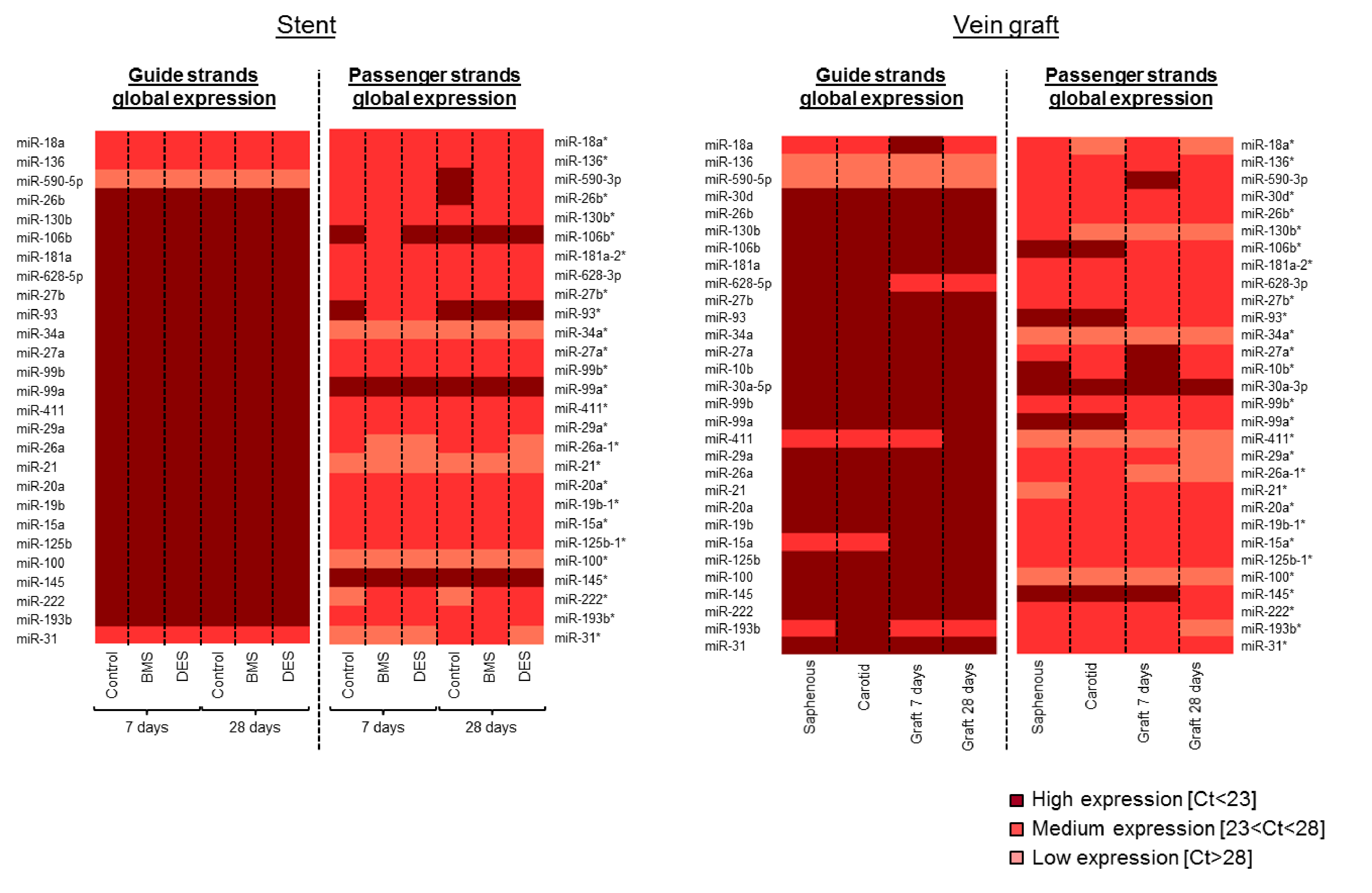

Supplement: Supplementary file 1 [file cells-08-00083-s001.zip › Pinel-Supplementary-files/Pinel-FigureS5.png]

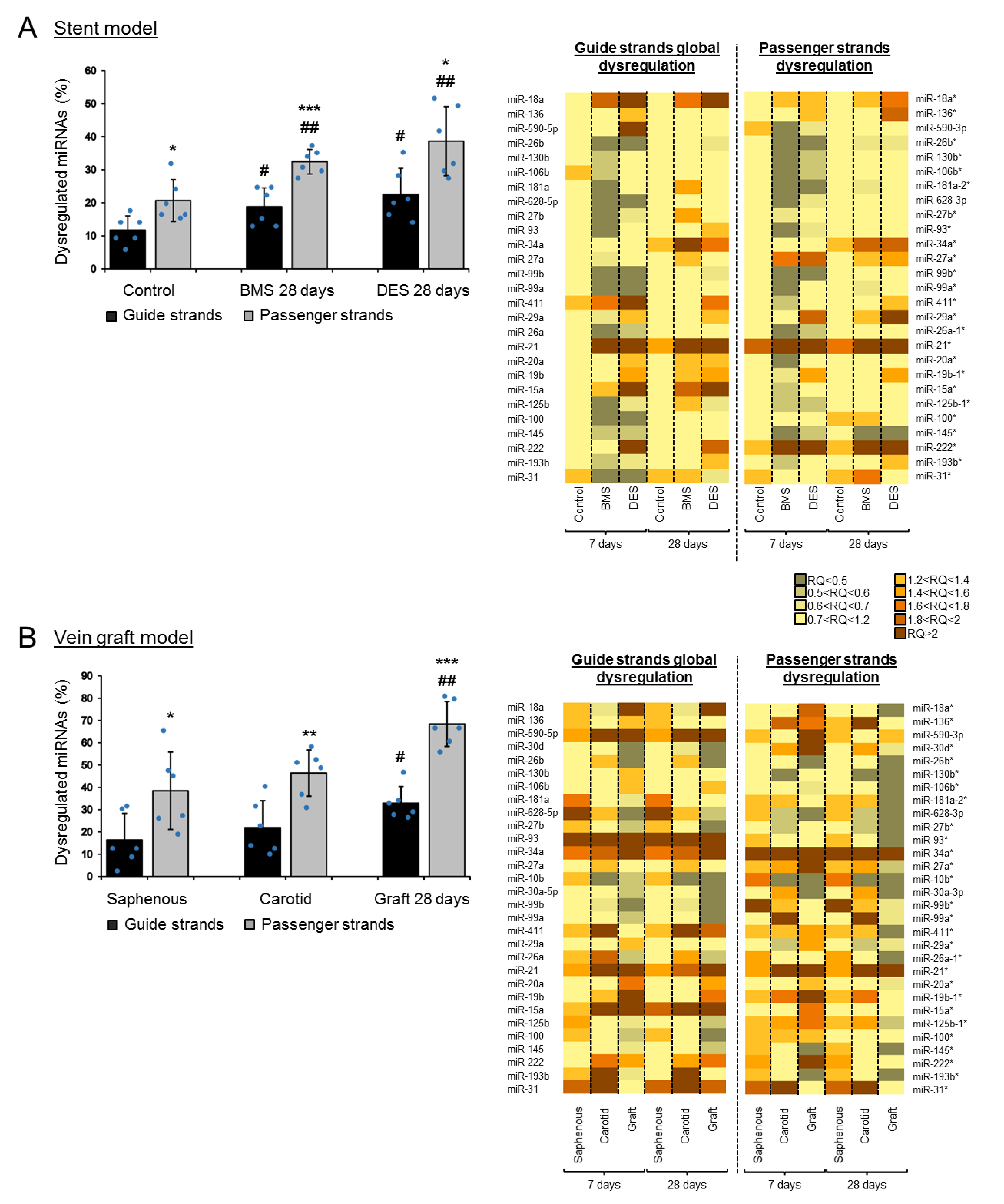

Supplement: Supplementary file 1 [file cells-08-00083-s001.zip › Pinel-Supplementary-files/Pinel-FigureS6.png]
